# Increased carotid intima–media thickness in pediatric nephrotic syndrome: A meta-analysis

**DOI:** 10.17305/bb.2025.12935

**Published:** 2025-09-18

**Authors:** Yongzheng Zhang, Mingda Song, Hai Wang, Lin Du

**Affiliations:** 1 The Second Department of Pediatrics, The First Affiliated Hospital, Heilongjiang University of Chinese Medicine, Harbin, China; 2 The Fourth Department of Cardiology, The First Affiliated Hospital, Heilongjiang University of Chinese Medicine, Harbin, China

**Keywords:** Nephrotic syndrome, carotid intima–media thickness, atherosclerosis, children, meta-analysis

## Abstract

Nephrotic syndrome (NS) in children has been associated with an increased risk of early atherosclerosis, as indicated by carotid intima–media thickness (cIMT). However, the existing literature on the relationship between NS and cIMT in pediatric populations presents inconsistent findings. This meta-analysis aims to compare cIMT measurements between children with NS and healthy controls. A comprehensive search of PubMed, Embase, and Web of Science was conducted through May 22, 2025. Observational studies that compared cIMT in children under 18 years with NS against controls were included. Mean differences (MDs) with 95% confidence intervals (CIs) were aggregated using a random-effects model to account for potential heterogeneity. Thirteen case-control studies involving 578 children with NS and 741 controls were analyzed. The results indicated that children with NS had significantly higher cIMT compared to controls (MD: 0.06 mm; 95% CI: 0.04–0.08; *P* < 0.001; *I*^2^ ═ 68%). Subgroup analyses revealed that the difference in cIMT was notably larger in studies with ≥60% male participants (MD: 0.09 mm) compared to those with <60% males (MD: 0.03 mm; *P* for subgroup difference = 0.01). No significant differences were observed based on age, disease duration, or adjustments for body mass index, blood pressure, or lipid profile (all *P* > 0.05). Meta-regression analyses suggested that the proportion of male participants and the rate of steroid-resistant NS (SRNS) may contribute to observed heterogeneity (adjusted *R*^2^ ═ 29.8% and 22.5%, respectively), although the slopes for these meta-regressions were not statistically significant (*P* ═ 0.13 and 0.87). In conclusion, children with NS exhibit increased cIMT compared to controls, indicating early vascular changes. The predominance of males and the presence of SRNS may partially account for the heterogeneity observed across studies.

## Introduction

Nephrotic syndrome (NS) is a prevalent chronic kidney disorder in children, characterized by significant proteinuria, hypoalbuminemia, hyperlipidemia, and edema [1, 2]. Diagnosis typically relies on clinical evaluation and laboratory tests that confirm nephrotic-range proteinuria and related biochemical abnormalities [3]. The annual incidence of pediatric NS ranges from 2 to 7 per 100,000 children, with increased prevalence noted in Asian populations [4]. Most cases are idiopathic and responsive to corticosteroid therapy [5, 6]; however, a substantial subset, particularly those with steroid-resistant NS (SRNS) or frequent relapses, necessitates prolonged immunosuppressive treatment [7, 8]. While kidney function is often preserved in the short term, NS and its treatments can have systemic repercussions, including elevated risks of infections [9–11], thromboembolism [12], and cardiovascular complications [13, 14]. Emerging evidence indicates that children with NS may experience early vascular alterations, even in the absence of overt cardiovascular disease [15], underscoring the necessity for sensitive markers of subclinical atherosclerosis in this demographic [16].

Carotid intima–media thickness (cIMT) is a non-invasive, ultrasound-based assessment of the thickness of the intimal and medial layers of the carotid artery wall [17]. It serves as a surrogate marker for subclinical atherosclerosis and has been demonstrated to predict future cardiovascular events in adults [18]. In pediatric populations, cIMT is increasingly acknowledged as an early indicator of vascular remodeling and endothelial dysfunction, particularly in individuals with chronic conditions, such as diabetes, obesity, and kidney disease [19, 20]. Several mechanisms may link NS to elevated cIMT, including persistent dyslipidemia, hypertension, systemic inflammation, endothelial injury, and oxidative stress—all of which may facilitate early atherogenesis [13, 15]. Despite heightened interest, studies assessing cIMT in children with NS have produced inconsistent findings, likely due to variations in sample size, disease duration, treatment status, and adjustments for confounding variables [21–33]. Consequently, this meta-analysis seeks to quantitatively synthesize available evidence comparing cIMT between children with NS and healthy controls, while also examining whether study-level characteristics—such as age, sex, disease duration, and steroid resistance—contribute to the observed heterogeneity.

## Materials and methods

This study adhered to the PRISMA 2020 [34, 35] and Cochrane Handbook guidelines [36] for conducting systematic reviews and meta-analyses, encompassing study design, data collection, statistical methods, and result interpretation. The protocol was registered in PROSPERO under ID CRD420251102090.

### Database search

To identify studies relevant to this meta-analysis, we conducted a comprehensive search of the PubMed, Embase, and Web of Science databases using an extensive array of search terms. This included the combined terms: (1) “carotid intima media thickness” OR “carotid intima–media thickness” OR “cIMT”; (2) “children” OR “pediatric” OR “paediatric”; and (3) “nephrotic syndrome” OR “nephrosis.” The search was limited to studies involving human subjects and included only full-length articles published in English in peer-reviewed journals. Additionally, we manually reviewed references from related original and review articles to identify further relevant studies. The search encompassed all records from database inception until May 22, 2025. The complete search strategy for each database is detailed in Supplemental data. We restricted our search to peer-reviewed English-language studies to ensure methodological rigor and data completeness; grey literature was excluded due to its often insufficient methodological detail for reliable meta-analysis. Search results from all databases were exported and consolidated in EndNote, with duplicate records automatically identified and manually verified prior to screening to ensure accurate study selection.

### Study eligibility criteria

We employed the PICOS framework to establish the inclusion criteria:

P (patients): Children (aged < 18 years) diagnosed with NS (including idiopathic, steroid-sensitive, or steroid-resistant types), irrespective of sex, ethnicity, or disease duration.

I (exposure): Diagnosis of NS, either active or in remission, serving as the primary exposure group.

C (comparison): Healthy controls or children without NS.

O (outcome): cIMT, measured via ultrasound as per the protocols of the original studies. Studies must report mean and standard deviation (or sufficient data to calculate these) for cIMT in both groups.

S (study design): Observational studies, including cross-sectional, case-control, or cohort studies.

Exclusions were applied to reviews, editorials, other meta-analyses, preclinical studies, studies lacking patients with NS or controls without NS, those involving adult patients, studies that did not assess cIMT, or those that did not report the data of interest. In cases of overlapping populations, we included the study with the largest sample size in the meta-analysis.

### Study quality evaluation

Two authors independently conducted the literature search, study selection, quality assessment, and data extraction. Inter-reviewer agreement for study selection and quality assessment was evaluated using Cohen’s κ statistic, with any disagreements resolved through discussion with the corresponding author. Study quality was assessed utilizing the Newcastle–Ottawa Scale (NOS) [37], which rates selection, control of confounders, and outcome evaluation. Scores range from 1 to 9, with scores of 7 or higher classified as good quality.

### Data collection

Data collected for analysis included study details (author, year, country, and design), patient characteristics (number of children with NS, duration of the disease, proportions of frequent relapsers and steroid-resistant NS), control characteristics (characteristics of controls and number of controls in each study), mean age, sex distribution, and mean body mass index (BMI) of the overall included children, methods for evaluating cIMT, and covariates matched or adjusted when examining the association between NS and cIMT in children.

### Statistical analysis

The difference in cIMT between children with NS and healthy controls was summarized as mean difference (MD) with a 95% confidence interval (CI) [36]. Heterogeneity was assessed using the Cochrane *Q* test and *I*^2^ statistic [38], where a *P* value < 0.10 indicated significant heterogeneity, and *I*^2^ values of <25%, 25%–75%, and >75% indicated low, moderate, and high heterogeneity, respectively. A random-effects model utilizing the DerSimonian–Laird (DL) approach was employed to pool the data, accommodating heterogeneity among studies [36]. To further validate the reliability of the results, a sensitivity analysis with the restricted maximum likelihood (REML) approach was also conducted [36]. τ^2^ was reported as generated by RevMan, which provides values with limited decimals and may round to 0.00; this limitation was acknowledged to avoid misinterpretation. Furthermore, we calculated 95% prediction intervals (PIs) for the primary analysis using the formula 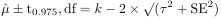, where *k* represents the number of datasets [39]. PIs estimate the likely range of effects in future comparable studies [39]. Sensitivity analyses were executed by excluding one study at a time. Additionally, sensitivity analysis was performed exclusively on studies with NOS ≥ 8. Predefined subgroup analyses were conducted based on mean patient ages, male proportions, disease duration, and other contributing factors to atherosclerosis, such as obesity (reflected as BMI), blood pressure (BP), or lipid profiles. Medians of continuous variables were utilized to evenly divide subgroups. Univariate meta-regression analysis was performed to investigate whether the difference in cIMT could be significantly influenced by study characteristics in continuous variables, including age, male proportion, disease duration, or the proportion of children with SRNS [36]. Multivariable meta-regression was not conducted due to the limited number of studies and non-significant findings in univariate analyses, which would hinder reliable estimation and risk overfitting. For outcomes involving at least 10 datasets, publication bias was assessed using funnel plots and visual inspection for asymmetry, supplemented by Egger’s test [40]. Potential small-study effects were further examined via the trim-and-fill method, which estimates the number of potentially missing studies due to publication bias and recalculates the pooled effect size after adjusting for these studies [36]. All analyses were executed using RevMan (Version 5.4; Cochrane Collaboration, Oxford, UK) and Stata (Version 17.0; Stata Corporation, College Station, TX, USA).

## Results

### Study inclusion

**Figure 1.**
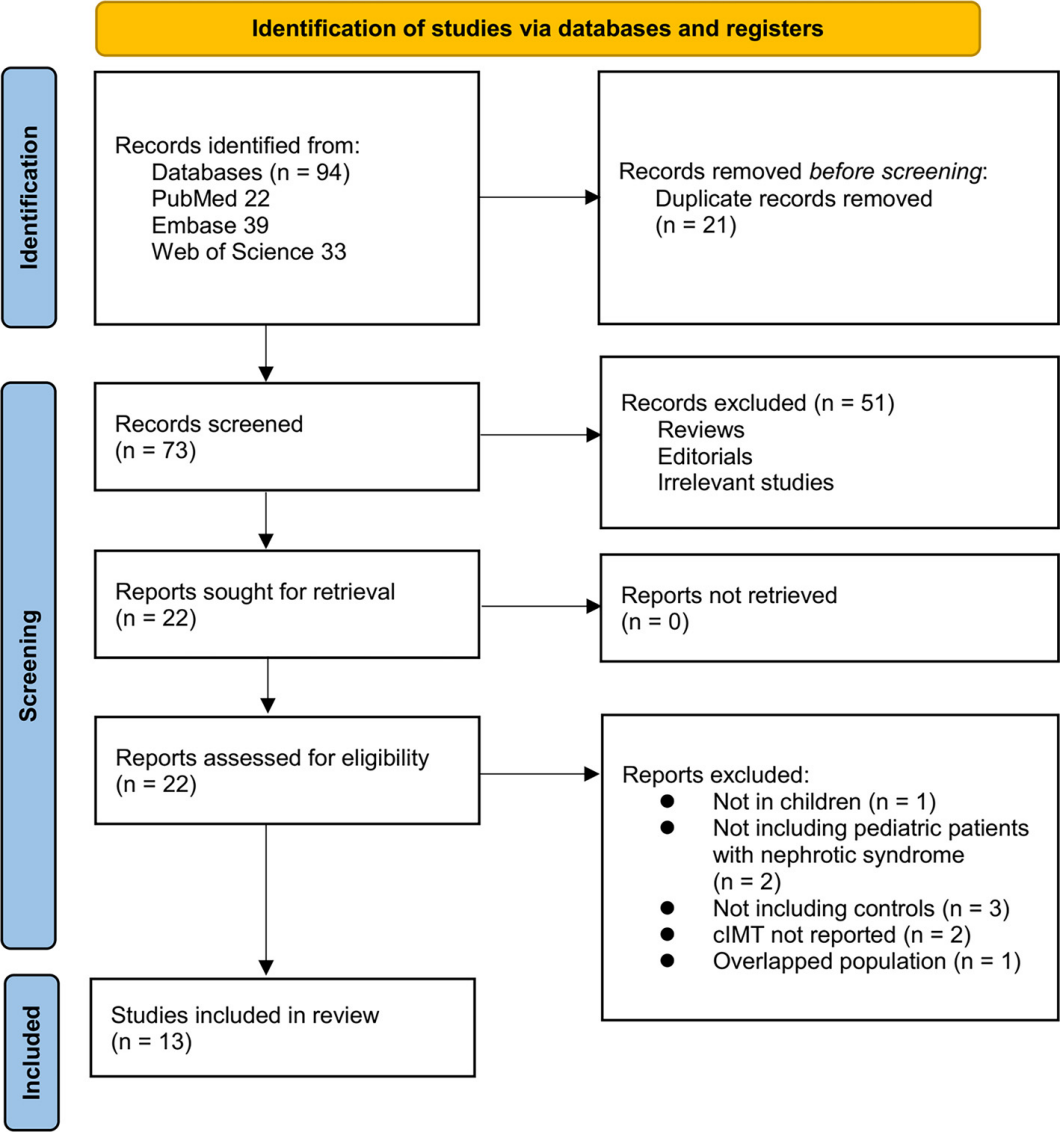
**Flowchart of database search and study inclusion.** The excluded record with overlapped population was Brachial Artery Flow-mediated Dilatation and Carotid Intima–Media Thickness in Children With Idiopathic Nephrotic Syndrome (Clin Exp Nephrol. 2015;19(1):125–132; PMID: 30595562), which overlapped with the cohort subsequently reported in Assessment of Atherosclerosis in Children with Nephrotic Syndrome (NeuroQuantology. 2022;20(6):8315–8328). The latter study with more complete data was retained.

The study selection process is illustrated in Figure 1. Initially, we identified 94 records from three databases. After removing 21 duplicates, 73 articles were screened based on their titles and abstracts. Out of these, 51 were excluded for not aligning with the objectives of the meta-analysis. The full texts of the remaining 22 articles were reviewed by two independent authors, leading to the exclusion of nine studies for various reasons (see Figure 1). Ultimately, 13 studies were included in the quantitative analysis [21–33]. The κ statistic for inter-reviewer agreement in study selection and quality assessment was 0.83, indicating a high level of consistency between reviewers.

**Table 1:** Characteristics of the included observational studies

**Study**	**Country**	**Design**	**No. of children with NS**	**Duration of NS (years)**	**Frequent relapser (%)**	**Steroid resistant (%)**	**Source of controls**	**No. of controls**	**Mean age (years) of overall children**	**Male (%) of overall children**	**Mean BMI of overall children (kg/m^2^)**	**Methods for evaluating cIMT**	**Variables matched or adjusted**
Hooman, 2013	Iran	CC	51	2.0	49.0	32.7	Children with history of UTI	75	7.9	58.7	17.2	B-mode ultrasound, average of bilateral	Age, sex, proteinuria, and SBP/DBP
Candan, 2014	Turkey	CC	37	7.9	NR	100.0	Healthy children	22	13.6	47.5	20.6	B-mode ultrasound, average of bilateral	Age, BMI, proteinuria, ferritin, and LDL-C
Rahul, 2015	India	CC	32	5.3	25.1	18.8	Healthy children	32	9.0	54.7	15.7	B-mode ultrasound, average of bilateral	Age, sex, BMI, and MDA
Kari, 2017	Saudi Arabia, Australia, UK	CC	8	3.4	NR	100.0	Healthy children	40	12.0	52.1	NR	B-mode ultrasound, average of bilateral	Age and sex
Skrzypczyk, 2019	Poland	CC	50	6.4	44.6	26.0	Healthy children	20	9.5	64.5	NR	B-mode ultrasound, average of bilateral	Age, sex, and BMI
Mehta, 2019	India	CC	66	2.4	24.2	4.5	Healthy children	128	7.5	59.7	NR	B-mode ultrasound, average of bilateral	Age, sex, BMI categories, and blood pressure
Paripovic, 2020	Serbia	CC	40	6.4	37.5	20.0	Healthy children	20	12.0	65.5	21.1	B-mode ultrasound, average of bilateral	Age, sex, BMI, and blood pressure
Ahmed, 2021	Egypt	CC	81	3.6	NR	13.7	Healthy children	100	8.2	68.5	16.8	B-mode ultrasound, average of bilateral	Age, sex, BMI, blood pressure, and lipid profile
Kamel, 2022	Egypt	CC	40	3.2	75.0	25.0	Healthy children	30	7.8	67.0	17	B-mode ultrasound, average of bilateral	Age, sex, BMI, hypertension, and dyslipidemia
Al Sharawy, 2022	Egypt	CC	20	1.0	20.0	20.0	Healthy children	25	9.0	60.0	NR	B-mode ultrasound, average of bilateral	Age, sex, hypertension, dyslipidemia
Das, 2024	India	CC	33	2.9	73.0	12.0	Healthy children	39	8.6	59.0	16.1	B-mode ultrasound, average of bilateral	Age, sex, BMI, and dyslipidemia
Elsehmawy, 2024	Egypt	CC	60	2.9	NR	36.7	Healthy children	60	9.7	65.0	20.7	B-mode ultrasound, average of bilateral	Age and sex
Esfandiar, 2024	Iran	CC	60	1.0	NR	NR	Healthy children	150	8.0	50.9	NR	B-mode ultrasound, average of bilateral	Age and sex

Abbreviations: NS: Nephrotic syndrome; CC: Case-control; UTI: Urinary tract infection; BMI: Body mass index; cIMT: Carotid intima–media thickness; SBP: Systolic blood pressure; DBP: Diastolic blood pressure; LDL-C: Low-density lipoprotein cholesterol; MDA: Malondialdehyde; NR: Not reported.

**Table 2:** Study quality evaluation via the Newcastle–Ottawa Scale

**Study**	**Adequate definition of cases**	**Representativeness of cases**	**Selection of controls**	**Definition of controls**	**Control for age and sex**	**Control for other confounders**	**Exposure ascertainment**	**Same methods for events ascertainment**	**Non-response rates**	**Total**
Hooman, 2013	1	1	1	1	1	1	1	1	1	9
Candan, 2014	1	1	1	1	0	1	1	1	1	8
Rahul, 2015	1	1	1	1	1	1	1	1	1	9
Kari, 2017	1	1	1	1	1	0	1	1	1	8
Skrzypczyk, 2019	1	0	1	1	1	1	1	1	1	8
Mehta, 2019	1	0	0	1	1	1	1	1	1	7
Paripovic, 2020	1	0	1	1	1	1	1	1	1	8
Ahmed, 2021	1	0	1	1	1	1	1	1	1	8
Kamel, 2022	1	1	1	1	1	1	1	1	1	9
Al Sharawy, 2022	1	1	1	1	1	1	1	1	1	9
Das, 2024	1	0	1	1	1	1	1	1	1	8
Elsehmawy, 2024	1	0	1	1	1	0	1	1	1	7
Esfandiar, 2024	1	1	1	1	1	0	1	1	1	8

### Summary of study characteristics

Table 1 presents the characteristics of the 13 studies included in this meta-analysis [21–33]. All studies employed a cross-sectional case-control design and were published between 2013 and 2024. These studies were conducted in various countries, including Iran, Turkey, India, Saudi Arabia, the UK, Poland, Serbia, and Egypt. For the four Egyptian studies [28–30, 32], we verified through the original reports that they were conducted at different centers with distinct recruitment periods and ethical approvals, ensuring the independence of the cohorts. The number of children with NS in each study ranged from 8 to 81 (total: 578), with disease durations spanning from 1.0 to 7.9 years. Several studies reported the proportion of frequent relapse cases (ranging from 20% to 75%) and steroid-resistant patients (4.5%–100%). Control groups predominantly consisted of healthy children, with one exception that included children with a history of urinary tract infection (UTI) [21]. Control sample sizes varied from 20 to 150 participants (total: 741). The mean age of participants across studies ranged from 7.5 to 13.6 years, and the percentage of male participants varied from 47.5% to 68.5%. Mean BMI was reported in eight studies [24–26, 29, 33], with means ranging from 15.7 to 21.1 kg/m^2^. The cIMT was assessed in all studies using B-mode ultrasound. Most studies measured the far wall of the common carotid artery with probe frequencies ranging from 5 to 13 MHz, while a few studies also included assessments of the carotid bulb or internal carotid artery (Table S1). Twelve studies adjusted for or matched age and sex [21, 23–33], while many also accounted for BMI, blood pressure, lipid profiles, or proteinuria to varying degrees [21, 23, 25–31]. Table 2 provides a quality assessment of the included studies based on the Newcastle–Ottawa Scale (NOS). Total NOS scores ranged from 7 to 9, indicating high methodological quality. Eleven studies achieved the maximum score of 8 or 9, reflecting robust case and control selection, appropriate confounder adjustment, and reliable exposure assessment [21–24, 26–31, 33]. The most commonly absent criterion was adjustment for confounders beyond age and sex.

### Difference in cIMT between children with NS and controls

**Figure 2.**
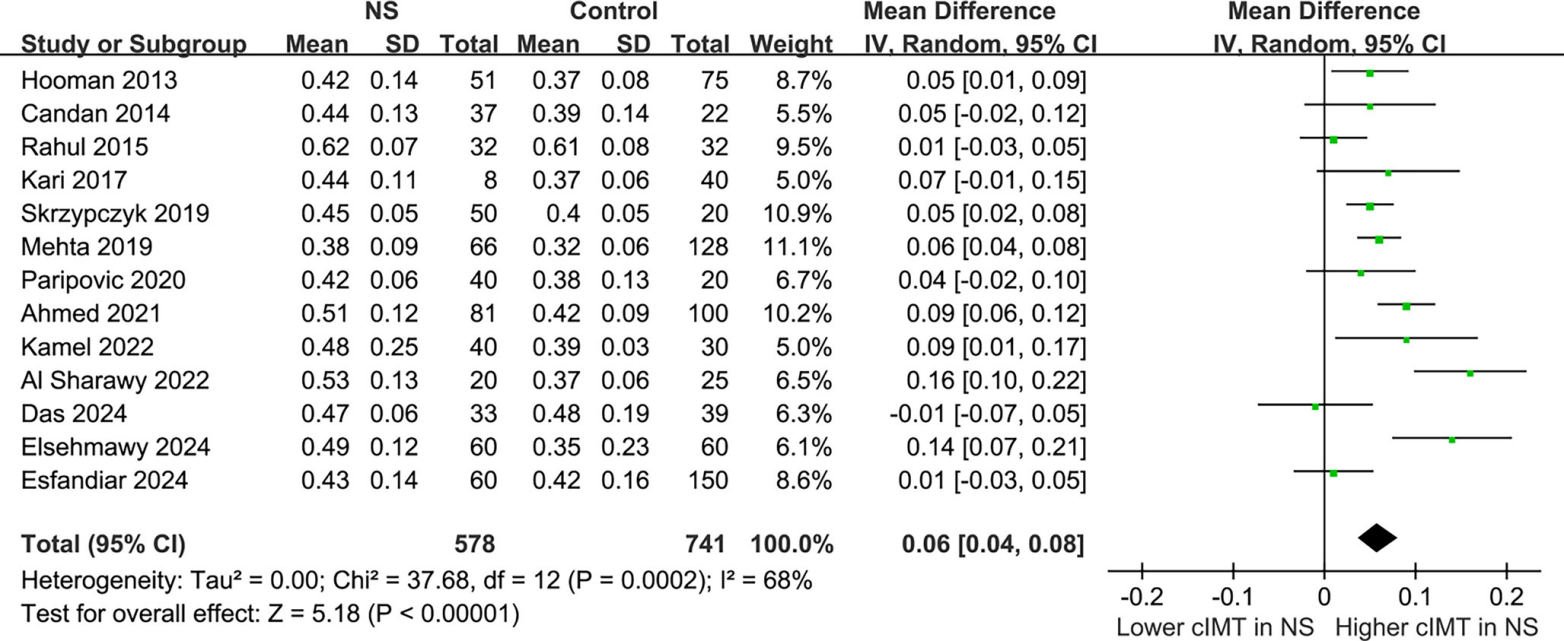
**Forest plots for the meta-analysis of the difference of cIMT between children with NS and controls.** Children with NS had significantly higher cIMT (MD: 0.06 mm, 95% CI: 0.04–0.08, *P* < 0.001) with significant heterogeneity (*I*^2^ ═ 68%). The 95% prediction interval was –0.01 to 0.13 mm. Abbreviations: cIMT: Carotid intima–media thickness; NS: Nephrotic syndrome; MD: Mean difference; CI: Confidence interval; *I*^2^: Inconsistency index.

**Figure 3.**
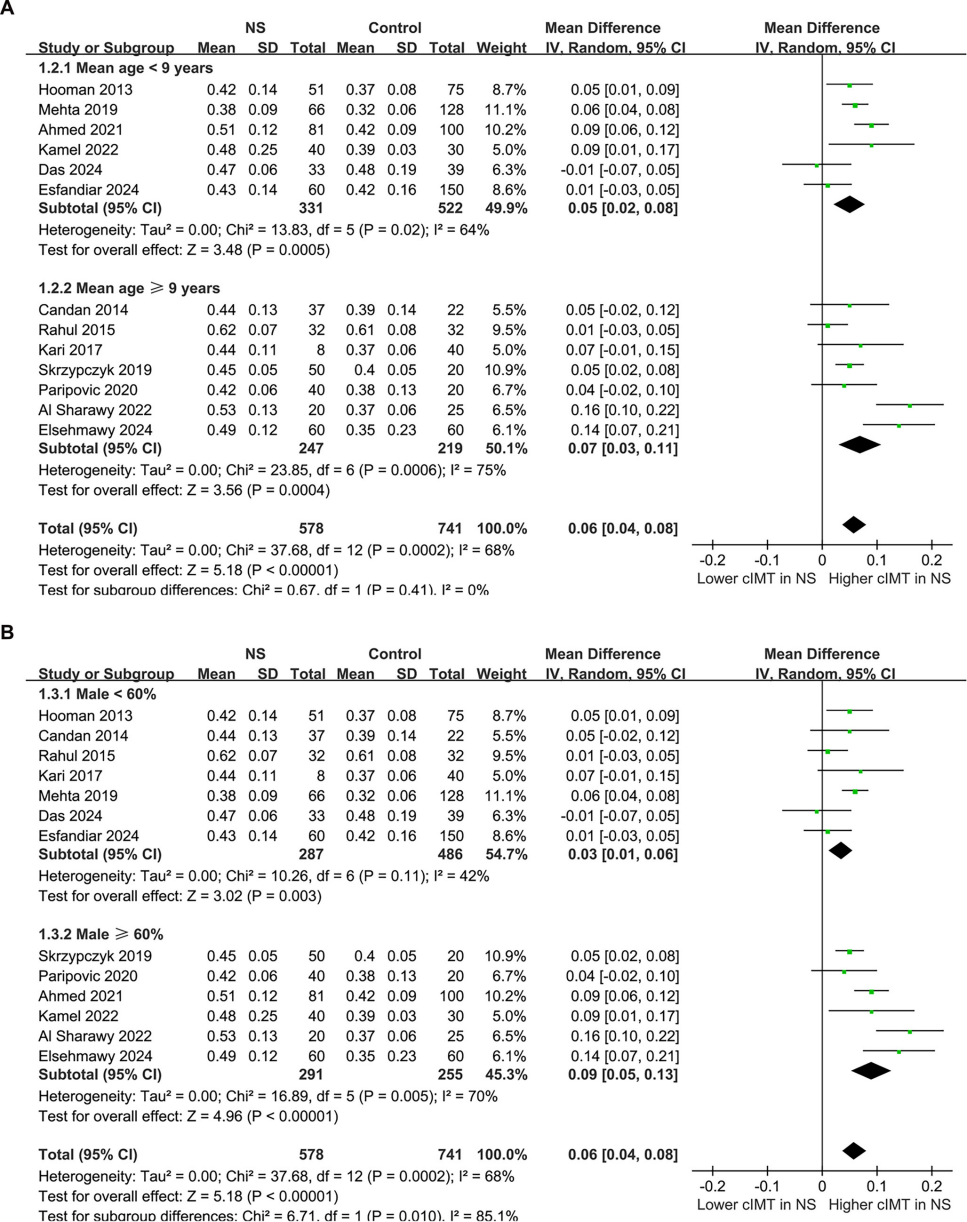
**Forest plots for the subgroup analyses of the difference of cIMT between children with NS and controls.** (A) Subgroup analysis according to the mean ages of the children; (B) Subgroup analysis according to the proportion of males. Abbreviations: NS: Nephrotic syndrome; SD: Standard deviation; CI: Confidence interval; IV: Inverse variance; cIMT: Carotid intima–media thickness.

**Figure 4.**
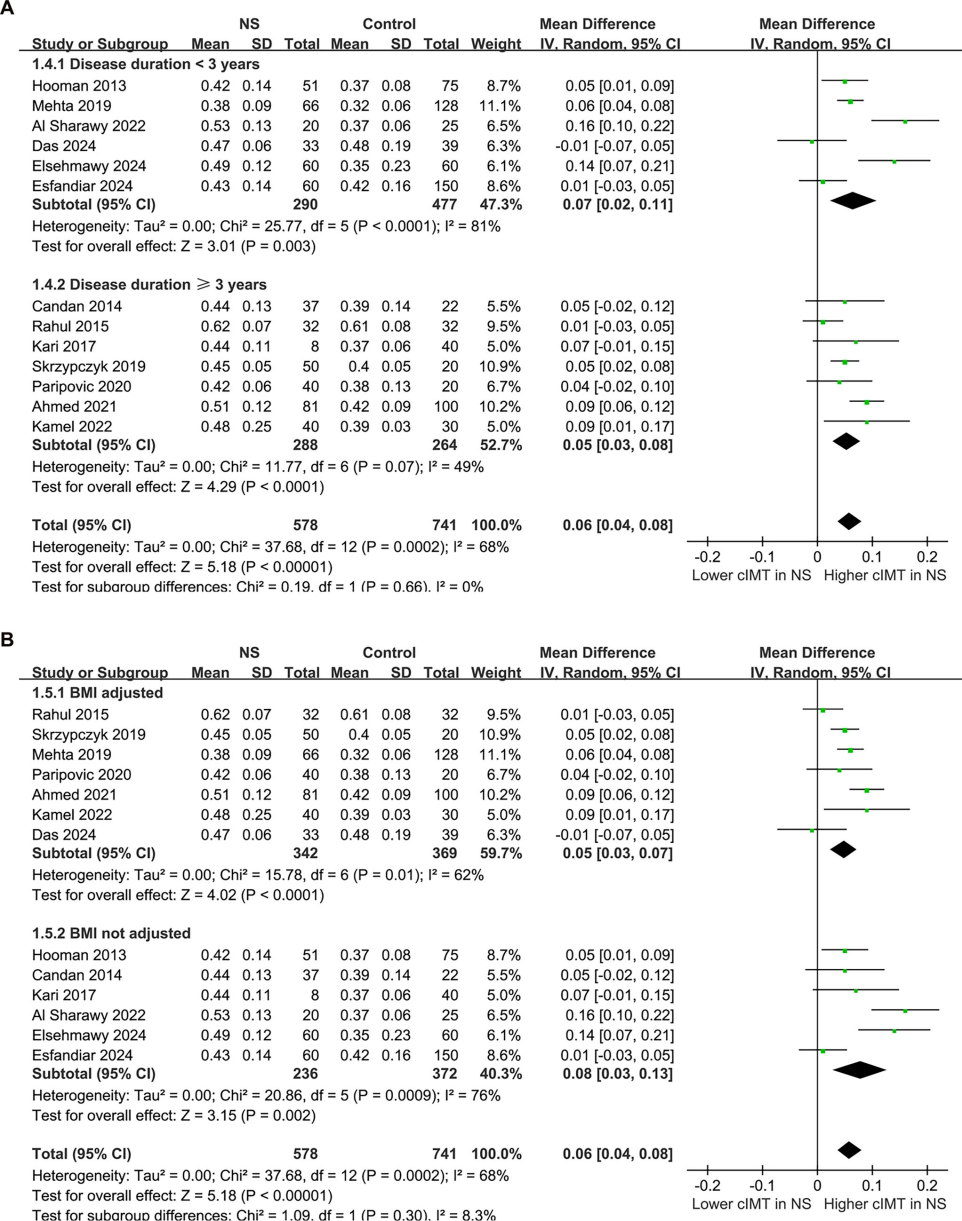
**Forest plots for the subgroup analyses of the difference of cIMT between children with NS and controls.** (A) Subgroup analysis according to the duration of NS; (B) Subgroup analysis according to whether BMI was adjusted. Abbreviations: NS: Nephrotic syndrome; SD: Standard deviation; CI: Confidence interval; IV: Inverse variance; cIMT: Carotid intima–media thickness; BMI: Body mass index.

**Figure 5.**
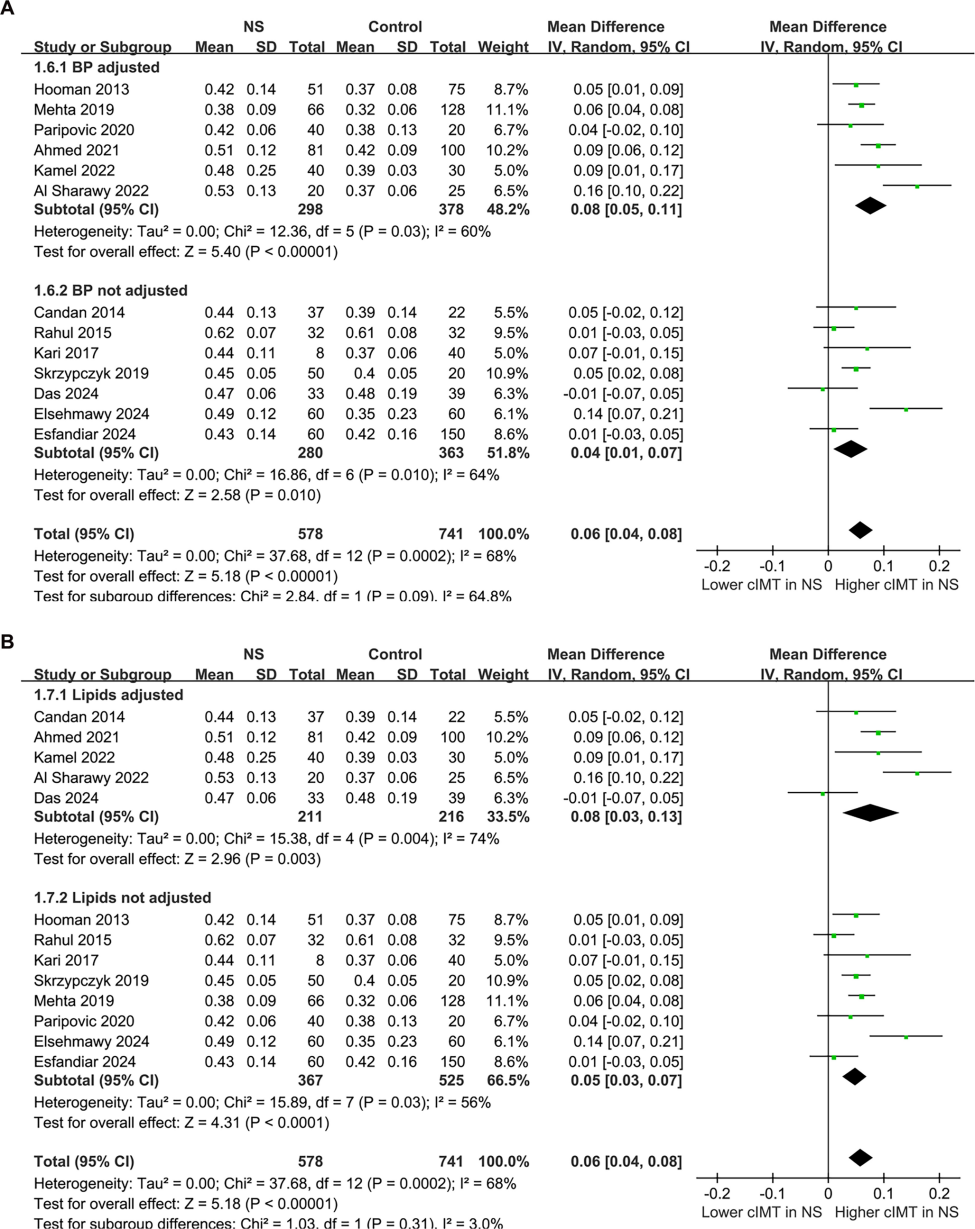
**Forest plots for the subgroup analyses of the difference of cIMT between children with NS and controls.** (A) Subgroup analysis according to whether BP was adjusted; (B) Subgroup analysis according to whether lipid profile was adjusted. Abbreviations: NS: Nephrotic syndrome; SD: Standard deviation; CI: Confidence interval; IV: Inverse variance; cIMT: Carotid intima–media thickness; BP: Blood pressure.

The pooled results from the 13 studies [21–33], analyzed using a random-effects model, indicated that children with NS exhibited a significantly higher cIMT compared to controls (MD: 0.06 mm, 95% CI: 0.04–0.08, *P* < 0.001; Figure 2), with notable heterogeneity (*P* for Cochrane *Q* test < 0.001, *I*^2^ ═ 68%). The 95% PIs for the primary analysis ranged from -0.01 to 0.13 mm, suggesting that while most future studies are anticipated to demonstrate a positive association, variability across settings could yield near-null effects. Additionally, a sensitivity analysis employing the REML approach yielded similar results (MD: 0.06 mm, 95% CI: 0.04–0.08, *P* < 0.001; *I*^2^ ═ 76%; Figure S1). Sensitivity analyses conducted by sequentially removing one study at a time, including each Egyptian dataset, yielded stable results (MD: 0.05–0.06 mm, *P* all < 0.05), indicating that no single study disproportionately influenced the overall estimate. Notably, the sensitivity analysis excluding the study that used children with a history of UTI as controls [21] produced similar outcomes (MD: 0.06 mm, 95% CI: 0.04–0.08, *P* < 0.001; *I*^2^ ═ 71%). Furthermore, the sensitivity analysis limited to studies with NOS scores ≥ 8 [21–24, 26–31, 33] also showed consistent results (MD: 0.05 mm, 95% CI: 0.03–0.08, *P* < 0.001; *I*^2^ ═ 68%). Subgroup analyses revealed comparable results for children < and ≥ 9 years (MD: 0.05 vs 0.07 mm, *P* for subgroup difference = 0.41; Figure 3A). Interestingly, a notably higher cIMT was observed in populations with a male proportion ≥60% compared to those with a male proportion <60% (MD: 0.09 vs 0.03 mm, *P* for subgroup difference = 0.01; Figure 3B). The results remained consistent for children with NS durations < and ≥ 3 years (MD: 0.07 vs 0.05 mm, *P* for subgroup difference = 0.66; Figure 4A). Further subgroup analyses indicated similar outcomes between studies with and without BMI adjustment (MD: 0.05 vs 0.08 mm, *P* for subgroup difference = 0.30; Figure 4B), blood pressure adjustment (MD: 0.08 vs 0.04 mm, *P* for subgroup difference = 0.09; Figure 5A), and lipid profile adjustment (MD: 0.08 vs 0.05 mm, *P* for subgroup difference = 0.31; Figure 5B). Finally, univariate meta-regression analysis results presented in Table 3 suggest that mean age, male proportion, disease duration, and SRNS proportion were not significant modifiers (all *P* > 0.05), although male proportion and SRNS might partially explain the observed heterogeneity (adjusted *R*^2^ ═ 29.8% and 22.5%, respectively). Given the limited number of studies and the associated power, these findings should be interpreted as exploratory rather than definitive.

**Table 3:** Results of univariate meta-regression analysis

**Variables**	**MD in the difference of cIMT between children with NS and controls**			
	**Coefficient**	**95% CI**	***P* values**	**Adjusted *R*^2^**
Mean age (years)	0.00030	−0.01721 to 0.01780	0.97	0%
Male (%)	0.0033	−0.0011 to 0.0076	0.13	29.8%
Duration of disease (years)	−0.0056	−0.0199 to 0.0086	0.41	0%
Steroid resistant (%)	0.000090	−0.001041 to 0.001220	0.87	22.5%

Abbreviations: MD: Mean difference; CI: Confidence interval; cIMT: Carotid intima–media thickness; NS: Nephrotic syndrome.

### Publication bias

**Figure 6.**
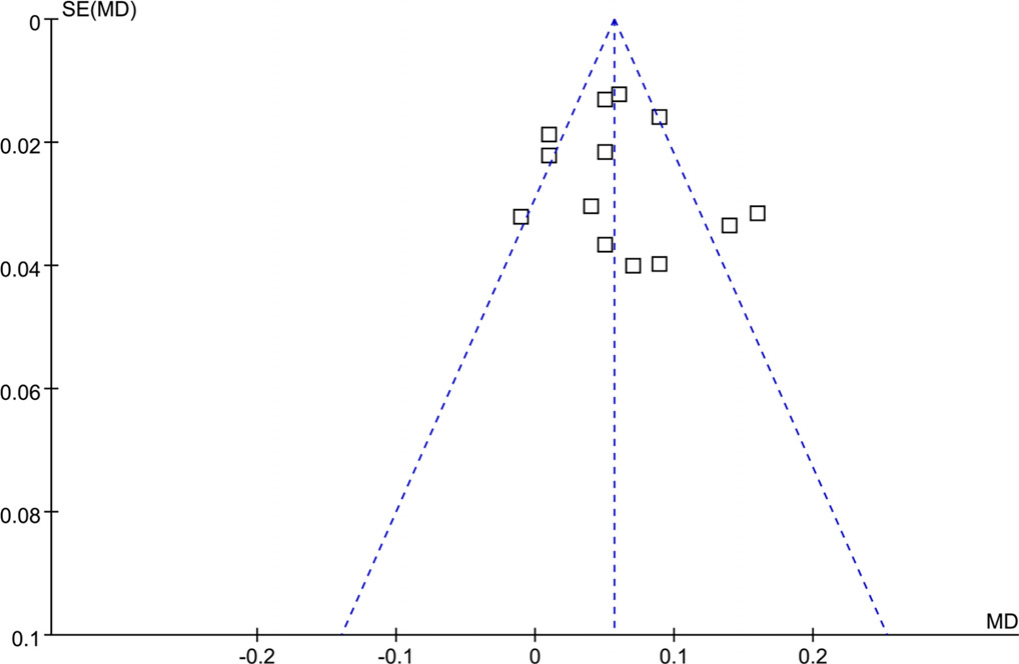
**Funnel plots for estimating the potential publication biases underlying the meta-analysis of the difference of cIMT between children with NS and controls.** Egger’s test *P* ═ 0.51. The plots appeared symmetrical, with no evidence of publication bias; the trim-and-fill method did not add studies, and the pooled MD remained unchanged. Abbreviations: NS: Nephrotic syndrome; cIMT: Carotid intima–media thickness; MD: Mean difference.

Funnel plots for the meta-analysis comparing cIMT between children with NS and controls are displayed in Figure 6. Although the plots appeared symmetrical and Egger’s test revealed no evidence of publication bias (*P* ═ 0.51), the statistical power of these tests is limited due to the small number of studies (*n* ═ 13). Furthermore, the trim-and-fill method did not impute additional studies, and the pooled MD remained largely unchanged (MD: 0.06 mm; 95% CI: 0.04–0.08; *P* < 0.001), suggesting that small-study effects are unlikely to have materially influenced the results.

## Discussion

This meta-analysis provides preliminary evidence that children with NS exhibit increased cIMT compared to their healthy peers, highlighting a potential association between NS and early vascular changes. While subclinical atherosclerosis has been extensively studied in adults with chronic kidney disease, our findings indicate that such vascular alterations may begin in childhood among patients with NS. This aligns with growing concerns regarding the long-term cardiovascular risks linked to chronic glomerular diseases and supports the integration of vascular health monitoring in pediatric nephrology. Additionally, our results suggest that specific clinical features, such as male predominance and a higher proportion of SRNS, may contribute to variability in cIMT outcomes across studies, identifying potential high-risk subgroups for early intervention.

Our findings are consistent with the recent 4C Study [41], which demonstrated a significant longitudinal increase in cIMT among children with chronic kidney disease and emphasized the impact of blood pressure dynamics on vascular progression. This underscores the necessity of early cardiovascular risk assessment in pediatric kidney disorders. Several mechanisms may contribute to increased cIMT in children with NS. From a pathophysiological standpoint, persistent proteinuria and hypoalbuminemia can lead to systemic inflammation and oxidative stress [42, 43], both of which contribute to endothelial dysfunction and arterial wall remodeling [44, 45]. Hyperlipidemia—a hallmark of NS—also plays a crucial role, as elevated low-density lipoprotein cholesterol (LDL-C) and triglycerides promote lipid deposition within vessel walls [46]. Furthermore, NS is frequently associated with hypertension and volume overload, which may increase shear stress and stimulate intima-media thickening [47]. Treatment-related factors, particularly prolonged use of corticosteroids or calcineurin inhibitors, may exacerbate cardiovascular risk by promoting insulin resistance, hypertension, or dyslipidemia [48]. Notably, recent studies have linked interleukin-6 polymorphisms with increased cIMT in children [49], supporting the hypothesis that inflammatory signaling pathways contribute to endothelial dysfunction and vascular remodeling in pediatric kidney disease. Clinically, repeated relapses and frequent exposure to immunosuppressive agents may further compound vascular stress [50]. These interconnected mechanisms provide a biologically plausible foundation for the observed association between NS and increased cIMT.

Our subgroup and meta-regression analyses provide crucial insights into sources of heterogeneity and potential effect modifiers. Notably, studies with ≥60% male participants exhibited a significantly greater difference in cIMT compared to studies with <60% males, indicating gender-based differences in vascular vulnerability or hormonal influences on endothelial function. This finding aligns with previous research suggesting that boys may experience adverse changes in vascular health earlier than age-matched girls [51]. Although not statistically significant, a similar trend was observed in studies with a higher proportion of SRNS patients, who typically endure more severe and treatment-resistant disease. Meta-regression analysis indicated that the male proportion and SRNS rate accounted for 29.8% and 22.5% of the between-study heterogeneity, respectively, highlighting their potential relevance despite limited power to detect significance. Conversely, subgroup analyses based on age, disease duration, and adjustments for BMI, blood pressure, or lipid profile revealed no significant differences. This suggests that the association between NS and cIMT may remain relatively consistent across various pediatric subgroups or that existing studies lacked sufficient granularity to detect these differences. Importantly, these subgroup analyses utilized study medians to maintain statistical balance, given the absence of universally accepted cutoffs for variables such as age or male proportion. Consequently, the findings should be interpreted as exploratory and hypothesis-generating rather than definitive.

The strengths of this meta-analysis include a comprehensive literature search across multiple databases, stringent inclusion criteria, and robust statistical methods, including sensitivity analyses, subgroup analyses, and meta-regression. All included studies employed ultrasound-based assessments of cIMT, ensuring methodological consistency in outcome measurement. Furthermore, the majority of studies were rated as high quality using the NOS, indicating a low risk of bias in study design and reporting. However, several limitations warrant acknowledgment. First, all included studies were cross-sectional, limiting conclusions about the temporal or causal relationships between NS and early vascular remodeling. Future prospective cohort studies or longitudinal ultrasound investigations are necessary to clarify whether increased cIMT precedes clinical cardiovascular events and to explore the dynamics of vascular changes over time. Second, while most studies adjusted for key confounders such as age and sex, the extent of adjustment for other cardiovascular risk factors—such as lipid levels, blood pressure, physical activity, and socioeconomic status—varied widely or was not detailed. Additionally, early-life [52] and maternal characteristics [53], including maternal health conditions, pregnancy complications, and birth parameters, are known to influence vascular phenotypes in offspring; however, these factors were inconsistently reported across included studies and could not be integrated into our analysis. Importantly, we did not compare the pooled cIMT difference with age-specific normative values or express it as a percentage of normative childhood cIMT due to the heterogeneity in age, ethnicity, and baseline vascular characteristics across included studies and the absence of standardized pediatric thresholds, which limits meaningful clinical interpretation of absolute cIMT differences. Third, analyses were based on aggregated study-level data rather than individual participant data, restricting the assessment of within-study interactions or detailed stratification by disease phenotype, treatment duration, or comorbidity burden. Moreover, subgroup cut points were median-based to maintain statistical balance across limited datasets rather than reflecting established clinical thresholds, which may affect the interpretability of subgroup differences. Fourth, moderate heterogeneity persisted despite subgrouping and regression analyses, suggesting that unmeasured factors may still influence the observed associations. The addition of a 95% PI (–0.01 to 0.13 mm) provides further context for between-study variability; while the lower bound approached the null, the interval remained centered on a positive effect, consistent with our pooled estimate. This suggests that most future studies would likely demonstrate increased cIMT in children with NS, although smaller or heterogeneous studies may yield attenuated associations. The width of the PI underscores the need for larger, prospective studies to refine effect estimates. Furthermore, although funnel plots and Egger’s test indicated no major publication bias, these methods have limited power with fewer than 20 studies, necessitating cautious interpretation of the results. Additionally, the small sample sizes of individual studies may have reduced the precision of effect estimates and limited the standardization of results across diverse populations. Our search was confined to PubMed, Embase, and Web of Science, the largest biomedical databases. While this approach adheres to PRISMA 2020 recommendations, it may have overlooked studies indexed exclusively in other platforms such as Scopus or EBSCO, introducing a small risk of selection bias. Furthermore, restricting the search to English-language, peer-reviewed articles and excluding grey literature may have omitted relevant studies, although this strategy is commonly employed to ensure methodological rigor and data reliability. Lastly, considerable heterogeneity in cIMT measurement protocols existed across studies, including variations in arterial segment selection, probe frequency, and caliper placement, which could not be accounted for in subgroup analyses due to incomplete reporting.

From a clinical perspective, even modest increases in cIMT during childhood may hold significant implications. In adult populations, cIMT differences as small as 0.01–0.02 mm have been associated with heightened cardiovascular risk [54]. In our analysis, the MD of 0.06 mm between children with NS and controls represents a potentially clinically significant early vascular change, particularly considering the young age of the studied populations [55]. These findings underscore the importance of cardiovascular risk monitoring in pediatric NS, particularly in patients with SRNS or those with prolonged disease courses. While adult studies suggest that small absolute increases in cIMT may carry prognostic significance, the clinical relevance of a 0.06 mm difference in children remains challenging to quantify. Due to heterogeneity in age, ethnicity, and measurement protocols across studies, we could not reliably express the pooled difference as a percentage of normative pediatric cIMT or in terms of standardized z-scores. This highlights the urgent need for large normative datasets and standardized pediatric reference values to enhance clinical interpretation. Clinicians should consider incorporating non-invasive vascular assessments, such as cIMT measurement, into routine follow-up protocols for high-risk children with NS. Moreover, the lack of standardized protocols and percentile-based reference values for pediatric cIMT assessment limits comparability across studies. Previous studies have emphasized the urgent need for the standardization of pediatric cIMT evaluation to facilitate reliable interpretation and clinical application [56]. Additionally, optimizing the management of modifiable cardiovascular risk factors—such as blood pressure, lipid levels, and obesity—should be a key priority in this population. Future research should prioritize prospective, longitudinal studies to clarify the temporal relationship between NS and vascular remodeling. Individual participant data meta-analyses or large cohort studies may assist in identifying which subgroups of children with NS are at the highest cardiovascular risk and in determining the clinical utility of cIMT monitoring in guiding therapeutic decisions. Furthermore, mechanistic studies are needed to explore the interplay between inflammation, endothelial dysfunction, and treatment effects in driving vascular changes. Interventional trials assessing the impact of lipid-lowering agents, antihypertensives, or lifestyle modifications on vascular outcomes in pediatric NS could also provide valuable clinical insights.

## Conclusion

In conclusion, this meta-analysis reveals that children with NS exhibit significantly elevated cIMT compared to control groups, indicating early vascular remodeling and an increased risk of cardiovascular complications. The association is notably stronger in populations with a higher male prevalence and among those with SRNS. These findings underscore the necessity for early cardiovascular evaluation and the management of risk factors in pediatric patients with NS, while highlighting the need for longitudinal studies to clarify the long-term clinical implications.

## Supplemental data

Supplemental data are available at the following link: https://www.bjbms.org/ojs/index.php/bjbms/article/view/12935/4007.

## Data Availability

All data generated or analyzed during this study are included in this published article.
